# Evaluation of European-based polygenic risk score for breast cancer in Ashkenazi Jewish women in Israel

**DOI:** 10.1136/jmg-2023-109185

**Published:** 2023-07-14

**Authors:** Hagai Levi, Shai Carmi, Saharon Rosset, Rinat Yerushalmi, Aviad Zick, Tamar Yablonski-Peretz, The BCAC Consortium, Qin Wang, Manjeet K Bolla, Joe Dennis, Kyriaki Michailidou, Michael Lush, Thomas Ahearn, Irene L Andrulis, Hoda Anton-Culver, Antonis C Antoniou, Volker Arndt, Annelie Augustinsson, Päivi Auvinen, Laura Beane Freeman, Matthias Beckmann, Sabine Behrens, Marina Bermisheva, Clara Bodelon, Natalia V Bogdanova, Stig E Bojesen, Hermann Brenner, Helen Byers, Nicola Camp, Jose Castelao, Jenny Chang-Claude, María-Dolores Chirlaque, Wendy Chung, Christine Clarke, NBCS Collaborators, Margriet J Collee, Sarah Colonna, CTS Consortium, Fergus Couch, Angela Cox, Simon S Cross, Kamila Czene, Mary Daly, Peter Devilee, Thilo Dork, Laure Dossus, Diana M Eccles, A. Heather Eliassen, Mikael Eriksson, Gareth Evans, Peter Fasching, Olivia Fletcher, Henrik Flyger, Lin Fritschi, Marike Gabrielson, Manuela Gago-Dominguez, Montserrat García-Closas, Jose Angel Garcia-Saenz, Jeanine Genkinger, Graham G Giles, Mark Goldberg, Pascal Guénel, Per Hall, Ute Hamann, Wei He, Peter Hillemanns, Antoinette Hollestelle, Reiner Hoppe, John Hopper, ABCTB Investigators, Simona Jakovchevska, Anna Jakubowska, Helena Jernström, Esther John, Nichola Johnson, Michael Jones, Joseph Vijai, Rudolf Kaaks, Elza Khusnutdinova, Cari Kitahara, Stella Koutros, Vessela Kristensen, Allison W Kurian, James Lacey, Diether Lambrechts, Loic Le Marchand, Flavio Lejbkowicz, Annika Lindblom, Sibylle Loibl, Adriana Lori, Jan Lubinski, Arto Mannermaa, Mehdi Manoochehri, Dimitrios Mavroudis, Usha Menon, AnnaMarie Mulligan, Rachel Murphy, Ines Nevelsteen, William G Newman, Nadia Obi, Katie O'Brien, Ken Offit, Andrew Olshan, Dijana Plaseska-Karanfilska, Janet Olson, Salvatore Panico, Tjoung-Won Park-Simon, Alpa Patel, Paolo Peterlongo, Brigitte Rack, Paolo Radice, Gad Rennert, Valerie Rhenius, Atocha Romero, Emmanouil Saloustros, Dale Sandler, Marjanka K Schmidt, Lukas Schwentner, Mitul Shah, Priyanka Sharma, Jacques Simard, Melissa Southey, Jennifer Stone, William J Tapper, Jack Taylor, Lauren Teras, Amanda E Toland, Melissa Troester, Thérèse Truong, Lizet E van der Kolk, Clarice Weinberg, Camilla Wendt, Xiaohong Rose Yang, Wei Zheng, Argyrios Ziogas, Alison M Dunning, Paul Pharoah, Douglas F Easton, Shay Ben-Sachar, Naama Elefant, Ron Shamir, Ran Elkon

**Affiliations:** 1 The Blavatnik School of Computer Science, Tel Aviv University, Tel Aviv, Israel; 2 Department of Human Molecular Genetics and Biochemistry, Tel Aviv University, Tel Aviv, Israel; 3 Braun School of Public Health and Community Medicine, The Hebrew University of Jerusalem, Jerusalem, Israel; 4 Department of Statistics and Operations Research, Tel Aviv University, Tel Aviv, Israel; 5 Institute of Oncology, Davidoff Cancer Center, Rabin Medical Center, Beilinson Hospital, Petah Tikva, Israel; 6 Sackler School of Medicine, Tel Aviv University, Tel Aviv, Israel; 7 Department of oncology, Hadassah Medical Center, Jerusalem, Israel; 8 Hebrew University of Jerusalem, Jerusalem, Israel; 9 Centre for Cancer Genetic Epidemiology, Department of Public Health and Primary Care, University of Cambridge, Cambridge, UK; 10 Biostatistics Unit, The Cyprus Institute of Neurology & Genetics, Nicosia, Cyprus; 11 Division of Cancer Epidemiology and Genetics, National Cancer Institute, National Institutes of Health, Bethesda, MD, USA; 12 Fred A. Litwin Center for Cancer Genetics, Lunenfeld-Tanenbaum Research Institute of Mount Sinai Hospital, Toronto, Ontario, Canada; 13 Department of Molecular Genetics, University of Toronto, Toronto, Ontario, Canada; 14 Department of Medicine, Genetic Epidemiology Research Institute, University of California Irvine, Irvine, CA, USA; 15 Division of Clinical Epidemiology and Aging Research, German Cancer Research Center (DKFZ), Heidelberg, Germany; 16 Oncology, Department of Clinical Sciences in Lund, Lund University, Lund, Sweden; 17 Translational Cancer Research Area, University of Eastern Finland, Kuopio, Finland; 18 Institute of Clinical Medicine, Oncology, University of Eastern Finland, Kuopio, Finland; 19 Department of Oncology, Cancer Center, Kuopio University Hospital, Kuopio, Finland; 20 Department of Gynecology and Obstetrics, Comprehensive Cancer Center Erlangen-EMN, Friedrich-Alexander University Erlangen-Nuremberg, Erlangen, Germany; 21 Division of Cancer Epidemiology, German Cancer Research Center (DKFZ), Heidelberg, Germany; 22 Institute of Biochemistry and Genetics, Ufa Federal Research Centre of the Russian Academy of Sciences, Ufa, Russia; 23 Department of Population Science, American Cancer Society, Atlanta, GA, USA; 24 Department of Radiation Oncology, Hannover Medical School, Hannover, Germany; 25 Gynaecology Research Unit, Hannover Medical School, Hamburg, Germany; 26 N.N. Alexandrov Research Institute of Oncology and Medical Radiology, Minsk, Belarus; 27 Copenhagen General Population Study, Herlev and Gentofte Hospital, Copenhagen University Hospital, Herlev, Denmark; 28 Department of Clinical Biochemistry, Herlev and Gentofte Hospital, Copenhagen University Hospital, Herlev, Denmark; 29 Faculty of Health and Medical Sciences, Copenhagen, Denmark; 30 German Cancer Consortium (DKTK), German Cancer Research Center (DKFZ), Heidelberg, Germany; 31 Division of Preventive Oncology, German Cancer Research Center (DKFZ) and National Center for Tumor Diseases (NCT), Heidelberg, Germany; 32 North West Genomics Laboratory Hub, Manchester Centre for Genomic Medicine, St Mary’s Hospital, Manchester University NHS Foundation Trust, Manchester Academic Health Science Centre, Manchester, UK; 33 Department of Internal Medicine and Huntsman Cancer Institute, University of Utah, Salt lake city, UT, USA; 34 Oncology and Genetics Unit, Instituto de Investigación Sanitaria Galicia Sur (IISGS), Xerencia de Xestion Integrada de Vigo-SERGAS, Vigo, Spain; 35 Cancer Epidemiology Group, University Cancer Center Hamburg (UCCH), University Medical Center Hamburg-Eppendorf, Hamburg, Germany; 36 CIBER of Epidemiology and Public Health (CIBERESP), Madrid, Spain; 37 Departments of Pediatrics and Medicine, Columbia University, New York, NY, USA; 38 Westmead Institute for Medical Research, University of Sydney, Sydney, New South Wales, Australia; 39 Department of Cancer Genetics, Institute for Cancer Research, Oslo University Hospital-Radiumhospitalet, Oslo, Norway; 40 Institute of Clinical Medicine, Institute of Clinical Medicine, Faculty of Medicine, University of Oslo, Oslo, Norway; 41 Department of Research, Vestre Viken Hospital, Drammen, Norway; 42 Section for Breast- and Endocrine Surgery, Department of Cancer, Division of Surgery, Cancer and Transplantation Medicine, Oslo University Hospital, Oslo, Norway; 43 Department of Radiology and Nuclear Medicine, Oslo University Hospital, Oslo, Norway; 44 Department of Pathology, Akershus University Hospital, Lorenskog, Norway; 45 Department of Tumor Biology, Institute for Cancer Research, Oslo University Hospital, Oslo, Norway; 46 Department of Oncology, Division of Surgery, Cancer and Transplantation Medicine, Oslo University Hospital-Radiumhospitalet, Oslo, Norway; 47 National Advisory Unit on Late Effects after Cancer Treatment, Oslo University Hospital, Oslo, Norway; 48 Department of Oncology, Akershus University Hospital, Lorenskog, Norway; 49 Oslo Breast Cancer Research Consortium, Oslo University Hospital, Oslo, Norway; 50 Department of Medical Genetics, Oslo University Hospital and University of Oslo, Oslo, Norway; 51 Department of Clinical Genetics, Erasmus Medical Center, Rotterdam, Netherlands; 52 Department of Computational and Quantitative Medicine, City of Hope, Duarte, CA, USA; 53 City of Hope Comprehensive Cancer Center, City of Hope, Duarte, CA, USA; 54 Department of Laboratory Medicine and Pathology, Mayo Clinic, Rochester, MN, USA; 55 Department of Oncology and Metabolism, Sheffield Institute for Nucleic Acids (SInFoNiA), University of Sheffield, Sheffield, UK; 56 Academic Unit of Pathology, Department of Neuroscience, University of Sheffield, Sheffield, UK; 57 Department of Medical Epidemiology and Biostatistics, Karolinska Institutet, Stockholm, Sweden; 58 Department of Clinical Genetics, Fox Chase Cancer Center, Philadelphia, PA, USA; 59 Department of Pathology, Leiden University Medical Center, Leiden, Netherlands; 60 Department of Human Genetics, Leiden University Medical, Leiden, Netherlands; 61 Nutrition and Metabolism Section, International Agency for Research on Cancer (IARC-WHO), Lyon, France; 62 Cancer Sciences, Faculty of Medicine, University of Southampton, Southampton, UK; 63 Channing Division of Network Medicine, Department of Medicine, Brigham and Women's Hospital and Harvard Medical School, Boston, MA, USA; 64 Department of Epidemiology, Harvard T.H. Chan School of Public Health, Boston, MA, USA; 65 Department of Nutrition, Harvard T.H. Chan School of Public Health, Boston, Massachusetts, USA; 66 Division of Evolution and Genomic Sciences, School of Biological Sciences, Faculty of Biology, Medicine and Health, University of Manchester, Manchester Academic Health Science Centre, Manchester, UK; 67 The Breast Cancer Now Toby Robins Research Centre, The Institute of Cancer Research, London, UK; 68 Department of Breast Surgery, Herlev and Gentofte Hospital, Copenhagen University Hospital, Herlev, Denmark; 69 School of Population Health, Curtin University, Perth, Western Australia, Australia; 70 Genomic Medicine Group, International Cancer Genetics and Epidemiology Group, Fundación Pública Galega de Medicina Xenómica, Instituto de Investigación Sanitaria de Santiago de Compostela (IDIS), Complejo Hospitalario Universitario de Santiago, SERGAS, Santiago de Compostela, Spain; 71 Medical Oncology, Hospital Clinico San Carlos, Madrid, Spain, USA; 72 Department of Epidemiology, Mailman School of Public Health, Columbia University, New York, NY, USA; 73 Herbert Irving Comprehensive Cancer Center, New York, New York, USA; 74 Cancer Epidemiology Centre, Cancer Council Victoria, Melbourne, Victoria, Australia; 75 Centre for Epidemiology and Biostatistics, Melbourne School of Population and Global Health, The University of Melbourne, Melbourne, Victoria, Australia; 76 Precision Medicine, School of Clinical Sciences at Monash Health, Monash University, Clayton, Victoria, Australia; 77 Department of Medicine, McGill University, Montreal, Quebec, Canada; 78 Division of Clinical Epidemiology, Royal Victoria Hospital, McGill University, Montreal, QU, Canada; 79 Team 'Exposome and Heredity', CESP, Gustave Roussy, INSERM, University Paris-Saclay, UVSQ, Villejuif, France; 80 Department of Oncology, Södersjukhuset, Stockholm, Sweden; 81 Molecular Genetics of Breast Cancer, German Cancer Research Center (DKFZ), Heidelberg, Germany; 82 Medical Oncology, Erasmus Medical Center, Rotterdam, Netherlands; 83 Dr Margarete Fischer Bosch Institute of Clinical Pharmacology, Stuttgart, Germany; 84 University of Tübingen, Tubingen, Germany; 85 Australian Breast Cancer Tissue Bank, Westmead Institute for Medical Research, University of Sydney, Sydney, New South Wales, Australia; 86 Research Centre for Genetic Engineering and Biotechnology 'Georgi D. Efremov', Skopje, North Macedonia; 87 Department of Genetics and Pathology, Pomeranian Medical University, Szczecin, Poland; 88 Independent Laboratory of Molecular Biology and Genetic Diagnostics, Pomeranian Medical University, Szczecin, Poland; 89 Department of Epidemiology and Population Health, Stanford University School of Medicine, Stanford, CA, USA; 90 Department of Medicine, Division of Oncology, Stanford Cancer Institute, Stanford University School of Medicine, Stanford, CA, USA; 91 Division of Genetics and Epidemiology, The Institute of Cancer Research, Sutton, UK; 92 Clinical Genetics Research Lab, Department of Cancer Biology and Genetics, Memorial Sloan Kettering Cancer Center, New York, NY, USA; 93 Clinical Genetics Service, Department of Medicine, Memorial Sloan Kettering Cancer Center, New York, NY, USA; 94 Department of Genetics and Fundamental Medicine, Bashkir State University, Ufa, Russia; 95 Radiation Epidemiology Branch, Division of Cancer Epidemiology and Genetics, National Cancer Institute, Bethesda, MD, USA; 96 Laboratory for Translational Genetics, Department of Human Genetics, KU Leuven, Leuven, Belgium; 97 VIB Center for Cancer Biology, VIB, Leuven, Belgium; 98 Epidemiology Program, University of Hawaii Cancer Center, Honolulu, HI, USA; 99 Clalit National Cancer Control Center, Carmel Medical Center and Technion Faculty of Medicine, Haifa, Israel; 100 Molecular Medicine and Surgery, Karolinska Institutet, Stockholm, Sweden; 101 Department of Clinical Genetics, Karolinska University Hospital, Stockholm, Sweden; 102 German Breast Group, GmbH, Neu Isenburg, Germany; 103 Institute of Clinical Medicine, Pathology and Forensic Medicine, University of Eastern Finland, Kuopio, Finland; 104 Biobank of Eastern Finland, Kuopio University Hospital, Kuopio, Finland; 105 Department of Medical Oncology, University Hospital of Heraklion, Heraklion, Greece; 106 MRC Clinical Trials Unit, Institute of Clinical Trials and Methodology, University College, London, UK; 107 Department of Laboratory Medicine and Pathobiology, University of Toronto, Toronto, Ontario, Canada; 108 Laboratory Medicine Program, University Health Network, Toronto, Ontario, Canada; 109 School of Population and Public Health, University of British Columbia, Vancouver, BC, Canada; 110 Cancer Control Research, BC Cancer Agency, Vancouver, BC, Canada; 111 Leuven Multidisciplinary Breast Center, Department of Oncology, Leuven Cancer Institute, University Hospitals Leuven, Leuven, Belgium; 112 Institute for Medical Biometry and Epidemiology, University Medical Center Hamburg-Eppendorf, Hamburg, Germany; 113 Epidemiology Branch, National Institute of Environmental Health Sciences, NIH, Research Triangle Park, NC, USA; 114 Department of Epidemiology, Gillings School of Global Public Health and UNC Lineberger Comprehensive Cancer Center, University of North Carolina at Chapel Hill, Chapel Hill, NC, USA; 115 Department of Quantitative Health Sciences, Division of Epidemiology, Mayo Clinic, Rochester, MN, USA; 116 Dipertimento Di Medicina Clinca e Chirurgia, Federico II University, Naples, Italy; 117 Genome Diagnostics Program, IFOM ETS - the AIRC Institute of Molecular Oncology, Milan, Italy; 118 Department of Gynaecology and Obstetrics, University Hospital Ulm, Ulm, Germany; 119 Unit of Molecular Bases of Genetic Risk and Genetic Testing, Department of Research, Fondazione IRCCS Istituto Nazionale dei Tumori (INT), Milan, Italy; 120 Centre for Cancer Genetic Epidemiology, Department of Oncology, University of Cambridge, Cambridge, UK; 121 Laboratorio de Oncología Molecular, Hospital Clínico San Carlos, Madrid, Spain; 122 Department of Oncology, University Hospital of Larissa, Larissa, Greece; 123 Division of Molecular Pathology, The Netherlands Cancer Institute, Amsterdam, Netherlands; 124 Division of Psychosocial Research and Epidemiology, The Netherlands Cancer Institute - Antoni van Leeuwenhoek hospital, Amsterdam, Netherlands; 125 Department of Clinical Genetics, Leiden University Medical Center, Leiden, Netherlands; 126 Department of Internal Medicine, Division of Medical Oncology, University of Kansas Medical Center, Westwood, KS, USA; 127 Genomics Center, Molecular Medicine, Université Laval, Quebec, Quebec, Canada; 128 Department of Clinical Pathology, The University of Melbourne, Melbourne, Victoria, Australia; 129 Genetic Epidemiology Group, School of Population and Global Health, University of Western Australia, Perth, Western Australia, Australia; 130 Epigenetic and Stem Cell Biology Laboratory, National Institute of Environmental Health Sciences, NIH, Research Triangle Park, NC, USA; 131 Department of Cancer Biology and Genetics, The Ohio State University, Columbus, OH, USA; 132 Family Cancer Clinic, The Netherlands Cancer Institute, Amsterdam, Netherlands; 133 Biostatistics and Computational Biology Branch, National Institute of Environmental Health Sciences, NIH, Research Triangle Park, NC, USA; 134 Department of Clinical Science and Education, Karolinska Institutet, Stockholm, Sweden; 135 Division of Epidemiology, Department of Medicine, Vanderbilt Epidemiology Center, Vanderbilt-Ingram Cancer Center, Vanderbilt University School of Medicine, Nashville, TN, USA; 136 Department of Computational Biomedicine, Cedars-Sinai Medical Center, West Hollywood, CA, USA; 137 Clalit Research Institute, Clalit Health Services, Ramat Gan, Israel; 138 Department of Genetics, Hadassah Medical Center, Jerusalem, Israel

**Keywords:** Genomics, Polymorphism, Genetic

## Abstract

**Background:**

Polygenic risk score (PRS), calculated based on genome-wide association studies (GWASs), can improve breast cancer (BC) risk assessment. To date, most BC GWASs have been performed in individuals of European (EUR) ancestry, and the generalisation of EUR-based PRS to other populations is a major challenge. In this study, we examined the performance of EUR-based BC PRS models in Ashkenazi Jewish (AJ) women.

**Methods:**

We generated PRSs based on data on EUR women from the Breast Cancer Association Consortium (BCAC). We tested the performance of the PRSs in a cohort of 2161 AJ women from Israel (1437 cases and 724 controls) from BCAC (BCAC cohort from Israel (BCAC-IL)). In addition, we tested the performance of these EUR-based BC PRSs, as well as the established 313-SNP EUR BC PRS, in an independent cohort of 181 AJ women from Hadassah Medical Center (HMC) in Israel.

**Results:**

In the BCAC-IL cohort, the highest OR per 1 SD was 1.56 (±0.09). The OR for AJ women at the top 10% of the PRS distribution compared with the middle quintile was 2.10 (±0.24). In the HMC cohort, the OR per 1 SD of the EUR-based PRS that performed best in the BCAC-IL cohort was 1.58±0.27. The OR per 1 SD of the commonly used 313-SNP BC PRS was 1.64 (±0.28).

**Conclusions:**

Extant EUR GWAS data can be used for generating PRSs that identify AJ women with markedly elevated risk of BC and therefore hold promise for improving BC risk assessment in AJ women.

**WHAT IS ALREADY KNOWN ON THIS TOPIC**

- Genome-wide association studies (GWASs) on breast cancer (BC) were, to date, mainly done on women of European (EUR) ancestry, and recent studies showed that polygenic risk score (PRS) based on these GWAS can effectively stratify EUR women according to their BC risk.
- However, PRS performance declines with the increase of the genetic distance between the population used in the GWAS and the population on which the PRS is applied.

**WHAT THIS STUDY ADDS**

- Here, we systematically evaluated the performance of EUR-based BC PRS on Ashkenazi Jewish (AJ) women from Israel. Our results demonstrate that extant EUR GWAS data can be used for generating PRSs that identify AJ women with markedly elevated risk of BC.

**HOW THIS STUDY MIGHT AFFECT RESEARCH, PRACTICE OR POLICY**

- Our study suggests the possibility of personalised BC screening programmes in Israel that could potentially improve early detection of BC while reducing overdiagnosis.

## Introduction

Breast cancer (BC) is the most common cancer diagnosed among women in Western countries including Israel, where some 5500 BC cases are diagnosed annually.^1^ An early diagnosis of BC leads to a higher cure rate and improved survival. Thus, it is essential to develop accurate risk prediction methods for identifying women at high risk of BC. An ongoing debate over the optimal approach to BC screening has led to discordant professional society recommendations.^2^ Two fundamental questions—whether to screen annually or at a lower frequency and whether screening should start at the age of 40 or at a later point in life—have been debated for over 20 years.^2–4^ In Israel, health providers generally recommend biennial mammography screening starting at age 50 for women, except for those with a family history of relevant cancer or carriers of pathogenic variants in BC-associated genes, who are recommended to start earlier and screen more frequently. This 'one size fits all' approach to nationwide BC screening might be suboptimal as it assumes an equal risk of developing BC to most women. A personalised screening strategy based on individual risk could enhance the early detection of BC, decrease the harm of overdiagnosis and unnecessary screens and improve the use of medical resources.^5^

Rare pathogenic variants in the *BRCA1* and *BRCA2* genes confer high risk of developing BC but account for only a small proportion (<10%) of BC cases in the general population.^6 7^ In contrast, numerous common BC susceptibility variants have been discovered over the last decade through genome-wide association studies (GWASs).^8 9^ Each of these variants confers only a small risk individually, but their combined effect, commonly estimated by a polygenic risk score (PRS), can be substantial.^10 11^ Importantly, recent studies on women of European (EUR) ancestry demonstrated that PRS models can effectively stratify women according to their BC risk. In particular, women in the top 1% of an optimised PRS model, based on 313 BC risk SNPs, have >4-fold elevated risk of developing BC compared with those in the middle quintile (40%–60%).^12^ This amounts to ~3.5% of BC incidence falling in this top percentile. In terms of absolute risk, women in the top 1% had a lifetime risk of 32.6%, similar to the risk conferred by pathogenic variants in some of the moderate-impact BC predisposition genes such as *ATM* and *CHEK2*.^13 14^ These results show that PRS models can be powerful BC risk predictors and hold a promise for improving BC prevention programmes and assisting in early diagnosis of BC. These advances have led to the launching of clinical trials in which prevention programmes are guided by novel personalised risk prediction models that integrate PRS information.^5^

Unfortunately, PRS performance declines substantially as the genetic distance increases between the *discovery population* (used in the GWAS) and the *target population* (on which the PRS is used).^15^ The decline in performance is due to differences in effect sizes, allele frequencies and linkage disequilibrium (LD) patterns between populations. Since the vast majority of the currently available GWAS was done on people of EUR ancestry, the clinical usefulness of PRS models in other populations is limited. The decline in PRS performance in non-EUR populations might aggravate disparities in clinical genetics care between ethnic groups.^15^ Several studies showed that BC PRS generated from EUR GWAS summary statistics (EUR BC PRS) has lower performance on non-EUR women (eg, African–Americans).^16 17^ Yet, some studies demonstrated that EUR BC PRS performance on Latin American women—a large group with variable levels of Indigenous American, EUR and African ancestries—was similar to its performance on women of EUR ancestry.^18^

The population in Israel is highly heterogeneous, with Ashkenazi Jews (AJ) being one of its largest ethnic group. Given the relatively low genetic distance between the EUR and AJ populations,^19 20^ we hypothesised that EUR BC PRS could be used to develop clinically relevant PRS models for AJ women in Israel. To that end, we used the massive genetic resource generated by the multinational Breast Cancer Association Consortium (BCAC),^8^ which also contains an Israeli cohort, to conduct a systematic evaluation of the predictive performance of EUR BC PRS models on Israeli AJ women. We demonstrate that an EUR BC PRS can be adjusted to the AJ population and identify women with markedly elevated BC risk (OR >2.0 for AJ women in the top 10% compared with the middle quintile). We substantiate these findings using an independent cohort of AJ Israeli women.

## Materials and methods

### BCAC dataset

We analysed 132 335 EUR women from the BCAC: 72 899 cases and 59 436 controls. In addition, the BCAC includes an Israeli cohort (BCINIS/BCAC cohort from Israel (BCAC-IL)) of 2161 women: 1437 cases and 724 controls. According to the ‘ethnOt’ field in the BCAC phenotype file, all the women in the BCAC-IL cohort are tagged as ‘Jewish Ashkenazi’. In addition, there are 73 samples in the EUR cohort that are tagged as AJ.

All samples analysed were genotyped using the OncoArray chip. In our analysis, we used an imputed version of the data provided by BCAC. The imputation was done against the 1000 Genomes Project imputation panel. In BCAC-IL, 119 (5.5%) *BRCA1/2* mutation carriers were identified by a self-reporting field provided by the BCAC.

### Hadassah Medical Center (HMC) cohort

The HMC dataset contains 181 Israeli AJ women, of whom 118 are BC cases under the age of 45 years and 63 are controls older than 75 years. We validated either by sequencing or genotyping that none of the women carried one of the three AJ founder mutations in *BRCA1/2*. Samples were genotyped using the Axiom PMDA chip. Likely pathogenic variants in selected genes are covered by this chip.Three women carried such variants in *BRCA1/2*, and none bore pathogenic variants in other BC susceptibility genes.

We phased the data using SHAPEIT2^21^ and imputed it using IMPUTE2.^22^ The imputation reference panel was generated using SHAPEIT2 from the EUR samples from the 1000 Genomes Project (n=503). Using PLINK, we filtered out SNPs with uncertainty greater than 0.1.

For the evaluation of the 313 PRS, we were able to map 304 SNPs, of which 248 were called (either by genotyping or imputation) in more than 90% of the samples.

### Quality check (QC) of discovery sets

We performed QC on each discovery set using PLINK.^23 24^ We kept only SNPs with minor alllele frequency (MAF) of ≥5%, HWE p value of ≥1e-6 and missing rate of ≤10%. In addition, we kept only samples where less than 10% of SNPs present in the set were missing. In addition, we filtered out ambiguous and duplicated alleles. A total of 4 617 515 SNPs remained in the BCAC-EUR cohort and 4 973 754 SNPs in the BCAC-EUR cohort after exclusion of the Polish samples.

Similarly, we used PLINK to perform QC on each target set. We kept the same HWE, missing rate and MAF thresholds as in the discovery set, filtered out duplicated alleles and kept samples where less than 10% of SNPs present in the set were missing. This process left 5 549 031 and 5 704 856 SNPs on the entire Israeli (BCAC-IL) and, used as a control, the entire Polish (BCAC cohort from Poland (BCAC-PL)) cohorts, respectively. Note that in cross-validation (CV) analyses (see further), to avoid information leakage, we performed QC on each fold separately, so the number of SNPs in each fold slightly varied, depending on the subset of individuals in the fold.

### GWAS analysis

We ran GWAS analyses for two sets: EUR (n=132 335) and EUR without the PL cohort (n=128 153). Both sets did not contain the BCAC-IL women. For each analysis, we ran PCA and GWAS using PLINK2 (with the --glm command)^24^ and generated GWAS summary statistics with the first five principal components as covariates.

### Nested CV

**Figure 1:**
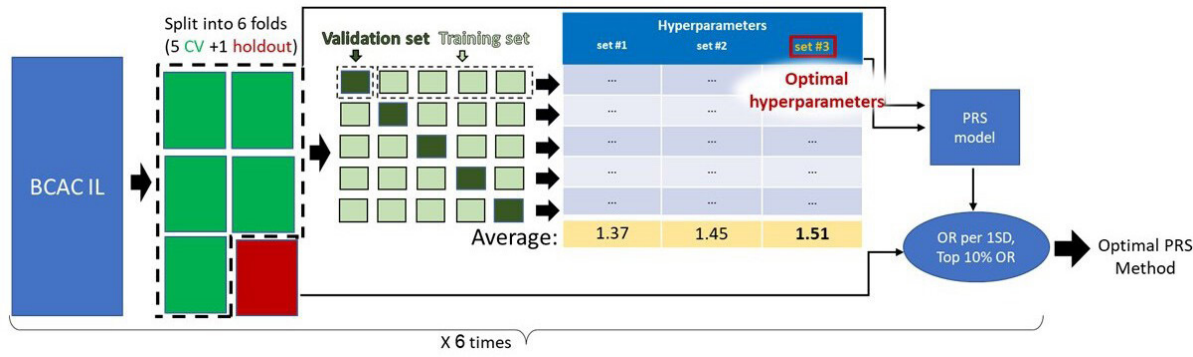
Outline of the CV scheme used to construct and evaluate the PRS models. We applied nested CV to optimise PRS models on the AJ cohort. Specifically, we split the BCAC-IL cohort into six sets (each of size 360). Next, we held out one set (red box) and used the other five sets (green boxes) to perform a standard fivefold CV in which four out of five parts (training set, light green) are used to derive PRS models with different predefined sets of hyperparameters (see the Materials and methods section), and then the resulting models are applied on the fifth part (validation set, dark green). For each PRS model, we measured the OR per 1 SD and the top 10% OR. After iterating over the five combinations of training and test sets, we chose the hyperparameter set with the highest average performance (see detailed ranking criteria in the Materials and methods section). Then, we retrained a PRS model on the five CV folds with the chosen hyperparameters. Finally, we applied the resulting PRS model on the holdout set and measured the OR per 1 SD and for the top 10% OR. We repeated this entire process six times, each with a different holdout set. We applied this scheme to each of the four PRS methods included in our analysis (P+T EUR-LD, P+T target LD, LDpred2 and Lassosum). The method that obtained the highest average performance on the six holdout sets is selected as the best one. AJ, Ashkenazi Jewish; BCAC, Breast Cancer Association Consortium; BCAC-IL, BCAC cohort from Israel; CV, cross validation; PRS, polygenic risk score; P+T EUR-LD, pruning and thresholding using European linkage disequilibrium; P+T target LD, pruning and thresholding using linkage disequilibrium of the target population.

We applied nested CV for optimising PRS models generated by four different methods (pruning and thresholding using European linkage disequilibrium (P+T EUR-LD), pruning and thresholding using linkage disequilibrium of the target population (P+T target set LD), LDpred2 and Lassosum; see futher). Specifically, for each PRS method, we split the BCAC-IL cohort into six sets (each of size 360). Next, we held out one set (red box in figure 1) and used the other five sets (green boxes in figure 1) to perform a standard 5-fold CV, in which four out of five parts (training set; light green) are used to derive PRS models with different predefined sets of hyperparameters, and then the resulting models are applied on the fifth part (validation set, dark green). For each model, we measured the OR per 1SD (using logistic regression with the first six principal components as covariates) and OR of women at the top 10% of the PRS distribution compared with the middle quintile. After iterating over the five combinations of training and test sets, we chose the hyper-parameter set that performed the best on average (see detailed ranking criteria below). Then, using these optimal hyper-parameters, we retrained a PRS model on the entire five CV folds (green boxes). Finally, we applied the resulting PRS model on the holdout set and measured the OR per 1SD and top 10% OR. We repeated this entire process six times, each with a different holdout set. The method with the highest average on the six holdout sets is nominated as the best one.

**Figure 2:**
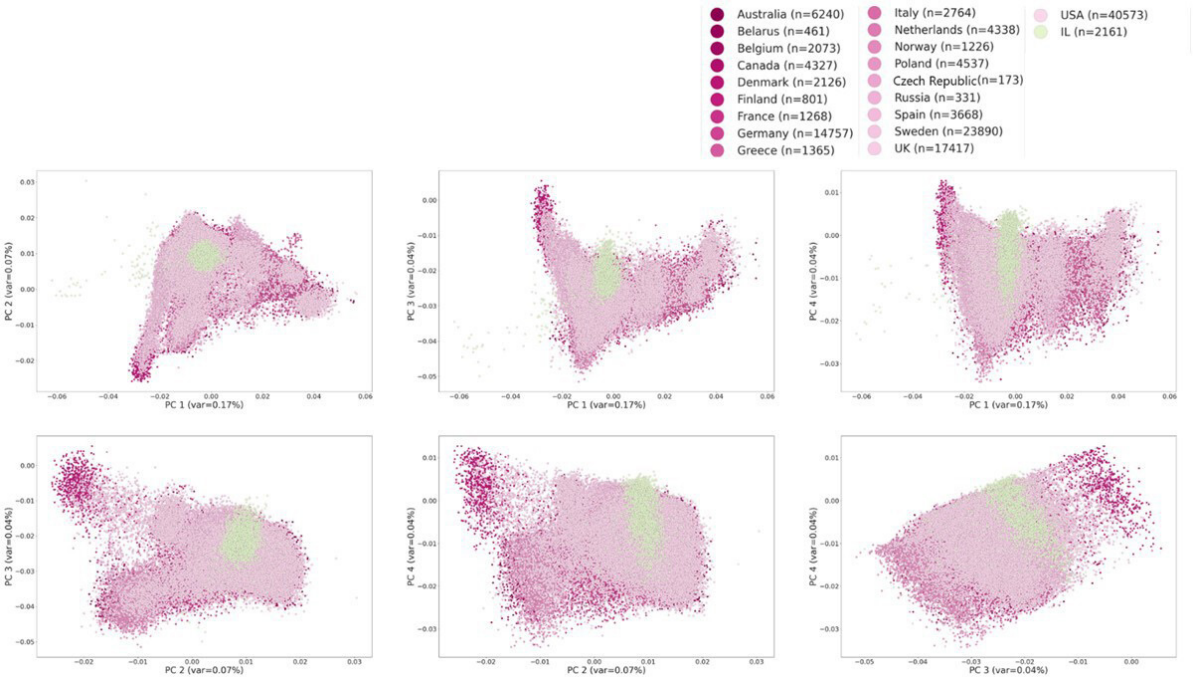
PCA on the EUR BCAC dataset. PCA was computed without BCAC-IL, which was later projected on it. Shown are two-dimesnional projections of PCs 1–4. The plot demonstrates high genetic similarity between the EUR and Israeli AJ populations. AJ, Ashkenazi Jewish; BCAC, Breast Cancer Association Consortium; EUR, European; BCAC-IL, BCAC cohort from Israel; PC, principal component; PCA, principal component analysis

In all analyses, PRS were standardised to the control samples of the respective target set.

### Criteria for choosing an optimal PRS model

We tested the performance of each PRS method with a predefined set of hyper-parameters (see below). For each method, we ranked runs with different hyper-parameters using two metrics: (1) OR per 1SD and (2) top-10% OR, and combined these rankings by taking their sum. We broke ties using the model with the higher OR per 1SD, as this metric is less noisy.

### Pruning and thresholding using European linkage disequilibrium

Using PLINK, we clumped the GWAS results according to LD in the EUR population derived from the EUR samples in the 1000 Genomes Project (n=503) with $r^{2}$ =0.2. Then, we filtered the remaining SNPs based on a significance threshold (T). We tested the following threshold values T:

$$5\cdot 10^{-8},\;10^{-7},\;10^{-6},\;10^{-5},10^{-4},10^{-3},\;5\cdot 10^{-3},10^{-2},5\cdot 10^{-2},0.1,\;0.2,\;0.3,\;0.4,\;0.5$$

For each T, we calculated the PRS from the SNPs that passed the filtering.

### Pruning and thresholding using linkage disequilibrium of the target population

Here, when applying LD clumping in PLINK, we used LD inferred from the training set. The training set comes from the same population as the target set. Namely, in each fold of the CV, LD was calculated using the genotype data of individuals in the training set. On the HMC cohort, we used the LD from the BCAC-IL cohort. The subsequent steps of the analysis are identical to the P+T EUR-LD method.

### LDpred2

LDpred2 (grid mode) generates a PRS model using SNP correlations calculated from genotype data (ie, the training set). We supplied LDpred2 with a training set that comes from the same population as the target set, as for the P+T method previoously. We ran LDpred2 using the set of hyper-parameter values for the proportion of causal variants, heritability, and sparseness that were recommended by.^25^ The rest of the hyper-parameters were left with their default values.

### Lassosum

Lassosum generates a PRS model using a reference panel calculated from genotype data (ie, the training set). We supplied Lassosum with a training set that comes from the same population as the target set, as above. We ran Lassosum using LD blocks option ‘EUR.hg19’ and the values of the regularisation hyper-parameter s that were recommended by.^25^ The rest of the hyper-parameters were left with their default values.

### 313-SNPs EUR BC PRS model

We downloaded the weights for the EUR PRS model from.^12^ Originally, the model consisted of 313 SNPs. In the imputed data, we managed to retain all the 313 SNPs for the BCAC-IL cohort and 304 SNPs for the HMC cohort. Risk scores for each sample were calculated using PLINK.

## Results

We set to build and evaluate EUR-based BC PRS for AJ women from Israel. For this task, we used an Israeli cohort of 2161 AJ women (1437 BC cases and 724 controls) that is a part of the BCAC (Methods). We refer to the Israeli sub-cohort of the BCAC as *BCAC-IL*. In order to avoid inflation of the predictive performance, the target set should be independent of the discovery set. Therefore, we could not reliably assess how the commonly used EUR BC 313-SNP PRS^12^ performs on the BCAC-IL cohort since this PRS was derived from BCAC GWAS, which included the BCAC-IL cohort. Therefore, we first removed the Israeli women from the EUR BCAC cohort and recomputed GWAS summary statistics using only data from the 132 335 non-Israeli EUR women (72 899 cases and 59 436 controls; Methods). A PCA on the BCAC genotype data confirmed the close genetic relatedness of AJ to the EUR population (figure 2).

Next, we set to adapt an EUR-based BC PRS for AJ women from Israel. We constructed PRS models from the GWAS we generated using four different methods: P+T^26 27^ EUR-LD; P+T using LD of the target (AJ) population (P+T target LD), LDpred2^28^ and Lassosum.^29^ We used two metrics to evaluate the models produced by these algorithms: (1) the OR per 1 unit SD and (2) the OR of women in the top 10% of the PRS distribution relative to those in the middle quantile (top 10% OR). We constructed and evaluated the PRS models using a nested CV scheme (see the Materials and methods section). The outline of our evaluation procedure is depicted in figure 1.

**Figure 3:**
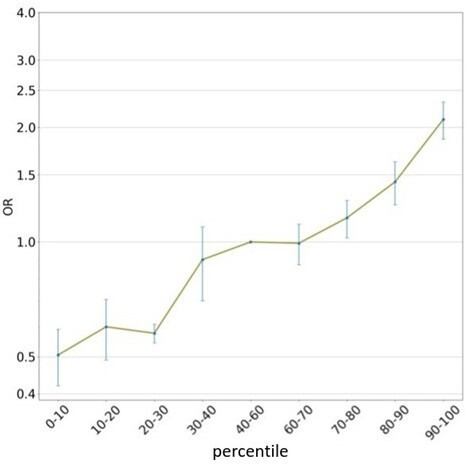
OR of BC risk as a function of BC PRS deciles. PRS was generated using Lassosum. OR is measured relative to scores in the middle PRS quintile (40%–60%). Shown are means and SEMs over the six holdout sets. BC, breast cancer; PRS, polygenic risk score.

Of the four methods we tested, Lassosum performed best, obtaining an OR per 1 SD of 1.56 (±0.09) and a top 10% OR of 2.1 (±0.24) (table 1 and online supplemental figure S1; see online supplemental table S1 for performance on the validation sets in the CV). We also examined the OR of other deciles of the PRS (compared with the middle quintile) and found that it increased nearly monotonically (figure 3). Further, women in the top 10% were estimated to have fourfold higher OR for BC compared with AJ women in the bottom 10% (figure 3, online supplemental figure S2). Notably, these top and bottom 10% OR estimates that we obtained for AJ women were comparable to those reported using EUR BC PRS on women of EUR ancestry.^12^

10.1136/jmg-2023-109185.supp1 Supplementary data

**Table 1:** Performance of different PRS methods on the BCAC-IL cohort

Method	OR per 1SD	Top 10% OR	SNPs (n)
P+T EUR-LD	1.43±0.08	1.43±0.27	1483±502
P+T target set LD	1.39±0.07	1.65±0.25	8591±5136
LDpred2	1.31±0.07	1.96±0.43	740 919±18
Lassosum	1.56±0.09	2.1±0.24	65 632±16 126

Performance of different PRS methods on the BCAC-IL cohort. ORs per 1 SD and top 10% OR were obtained using the nested CV outlined in figure 1. The last column is the average number of SNPs. Shown are means and SEMs over the six holdout sets.

BCAC-IL, BCAC cohort from Israel; CV, cross validation; PRS, polygenic risk score; P+T EUR-LD, pruning and thresholding using European linkage disequilibrium; P+T target set LD, pruning and thresholding using linkage disequilibrium of the target population.

Next, to estimate the decline in the performance of EUR-based BC PRS when applied to AJ women relative to women of EUR ancestry, we compared the performance obtained on women from BCAC-IL and women from BCAC-PL. We compared BCAC-IL to the Polish cohort as the AJ population is mainly from Eastern Europe. Specifically, we now excluded the Polish and Israeli samples from the BCAC discovery set and reran a GWAS analysis (see the Materials and methods section). Then, we applied the same nested CV scheme to the BCAC-PL (4537 women: 2318 cases and 2219 controls) and BCAC-IL cohorts using the same four PRS methods as previously discussed. As expected, the results obtained on BCAC-PL were mostly higher than those on BCAC-IL, reflecting the greater genetic distance of the AJ population from the EUR population (table 2; see online supplemental table S2 for performance on the validation sets).

**Table 2:** Performance of EUR PRS when excluding the Polish and Israeli cohorts from the discovery set and using these respective populations as the target cohorts

Method	Target set cohort	OR per 1 SD	Top 10% OR
P+T (EUR)	BCAC-IL	1.37±0.06	2.41±0.76
	BCAC-PL	1.46±0.06	2.2±0.15
P+T target LD	BCAC-IL	1.36±0.03	1.64±0.17
	BCAC-PL	1.5±0.07	1.85±0.16
LDPred	BCAC-IL	1.31±0.1	1.56±0.17
	BCAC-PL	1.17±0.06	1.82±0.33
Lassosum	BCAC-IL	1.50±0.06	1.82±0.3
	BCAC-PL	1.53±0.05	3.11±0.45

Shown are average ORs per 1 SD and top 10% ORs. Errors were measured using SEM for the six holdout sets.

BCAC-IL, BCAC cohort from Israel; BCAC-PL, BCAC cohort from Poland; P+T, pruning and thresholding; P+T target LD, pruning and thresholding using linkage disequilibrium of the target population.

Pathogenic variants in *BRCA1/2* confer a very high risk of BC. In BCAC-IL, 119 women were flagged as carriers of the *BRCA1/2* mutation (106 cases and 13 controls). To test the impact of the inclusion of these *BRCA1/2* carriers on PRS performance, we measured the performance of the P+T EUR-LD PRS on the BCAC-IL cohort after excluding these 119 samples. As shown in online supplemental figure S3, there was no significant difference between the two runs in the estimates for the OR per 1 SD and the top 10% OR.

To further examine the performance of EUR-based BC PRS on AJ women in Israel, we genotyped an independent sample of 181 Israeli AJ women recruited at the HMC in Jerusalem. This cohort comprises 118 patients with BC and 63 healthy women as controls. All the patients in the HMC cohort were diagnosed with BC at an early age (<45 years old) and tested negative for the three AJ founder variants in *BRCA1/2*. The controls were women aged 75 years and over who were never diagnosed with cancer. We first evaluated how the EUR BC 313-SNP PRS (313 PRS)^12^ performs on this cohort. Notably, the OR per 1 SD of the 313-PRS model was 1.64±0.28 on the HMC cohort, similar to the effect reported for this PRS model on EUR women (1.65 OR per 1 SD, 95% CI 1.59 to 1.79)^12^. For comparison, we also measured the performance of the 313 PRS on the BCAC-IL cohort and obtained OR per 1 SD of 1.77±0.09. This result is likely inflated due to the inclusion of the BCAC-IL in the discovery set used to infer the 313-PRS model. On the other hand, the OR estimate for the BCAC-IL cohort was less noisy than the one obtained in the HMC cohort due to its larger size (the BCAC-IL cohort is >10 times larger than the HMC).

Last, we evaluated Lassosum—the best performing method on BCAC-IL—on HMC. Using the EUR GWAS we generated, we trained the PRS model on the BCAC-IL cohort in fivefold CV (online supplemental figure S4). Applying this PRS to the HMC cohort yielded an OR of 1.58±0.27 per 1 SD (number of SNPs: 4540).

Overall, the results obtained on the HMC cohort reaffirm that EUR-based BC PRS has clinically relevant predictive capacity for Israeli AJ women.

## Discussion

PRS models have the potential to play an essential role in detecting women’s risk of developing BC. Nevertheless, at present, clinically relevant BC PRS models have been constructed primarily for women of EUR ancestry, for whom large discovery sets are currently available.^15^ Whether these models perform well on women of other ancestries and how they can be adapted for women of other ancestries are key open questions. Our study focuses on a major ethnic group in Israel, the Ashkenazi Jewish (AJ) population, which is genetically close to the EUR population. We tested whether a large number of available EUR genotypes of patients with BC and healthy women could be used to generate a clinically relevant BC PRS model for AJ women in Israel.

We evaluated four PRS methods on the Israeli cohort from BCAC (BCAC-IL) and found that Lassosum had the best prediction performance. Notably, there was a fourfold increased BC risk between women in the top and bottom 10% of the PRS distribution (figure 3 and online supplemental figure S2), suggesting that BC PRS models derived from EUR GWAS may help fit personalised recommendations for BC preventive screening for Israeli AJ women. The results obtained on the independent HMC cohort further support this conclusion. While the BCAC-IL cohort is too small to calculate reliable risk estimates for women in the top 5% and 1%, the monotonic increase of the OR with the deciles (figure 3) and results by similar BC PRS on EUR women^12^ suggest that this model has the capacity to identify at its very top percentiles AJ women with even higher risk of developing BC. Follow-up studies with larger samples of AJ women are needed to substantiate this expectation.

Notably, the HMC cohort has extreme age differences between the case and control arms: healthy women are older than 75 and patients with BC are younger than 45. Thus, the high prediction performance of the BC PRS models on this cohort suggests that EUR-based PRS models may also be relevant for detecting early-onset cases of BC among Israeli AJ women. In addition, these results indicate that for AJ women, low-impact common genetic variants—and not only pathogenic variants with high and moderate impact—play an important role in predisposing women to early-onset BC.

One limitation of our study is that *BRCA1/2* carriers were identified in the BCAC-IL only by self-reporting. Thus, there might be additional women carrying *BRCA1/2* variants who were marked as non-carriers as identified by.^30^ Still, our analysis indicates that inclusion of a limited group of patients who carry pathogenic variants in *BRCA1/2* genes does not have a significant impact on the PRS performance (online supplemental figure S3).

As the patients with BC at HMC were under 45, we could not directly generalise the prediction performance obtained on HMC for older AJ Israeli patients. However, online supplemental figure S5 indicates that there is no substantial difference in the PRSs between age groups of BCAC-IL patients, consistent with previous findings on EUR population.^12^

Our finding indicates that the currently available EUR BC GWAS data can be used to generate BC PRS models for Israeli AJ women. Nevertheless, this observation should not nullify the effort to genotype a higher number of individuals in Israel. First, an increased sample of AJ women would provide more accurate risk estimates for women at the top tail of the PRS distribution. Second, the Israeli population is highly heterogeneous, comprising many different ethnic groups, including North African and Middle Eastern Jews, as well as Palestinians, Druzes and Bedouins. Moreover, many of the younger generation in Israel are of mixed ethnicities. Therefore, to cover additional groups in nationwide BC prevention programmes, large-scale genotyping initiatives should include women from other ethnic groups in Israel, including admixed groups. Such data would allow a systematic evaluation of EUR-derived PRS BC models on non-AJ Israeli populations. We hope that this study will expedite the realisation of the potential for personalised BC risk stratification and encourage the development of screening protocols for high-risk women.

## Data Availability

Data are available upon reasonable request. The Breast Cancer Association Consortium (BCAC) data are available upon request from Cambridge University (see the BCAC website: https://bcac.ccge.medschl.cam.ac.uk/bcacdata/).The Hadassah Medical Center data are available from the corresponding author upon a reasonable request.

## References

[1] Ministry of Health . Breast cancer in women in Israel, update of morbidity and mortality data. 2022. Available: https://www.gov.il/en/departments/news/23102022-01

[2] Esserman LJ , WISDOM Study and Athena Investigators . The WISDOM study: breaking the deadlock in the breast cancer screening debate. NPJ Breast Cancer 2017;3:34. 10.1038/s41523-017-0035-5 28944288 PMC5597574

[3] Román M , Sala M , Domingo L , et al . Personalized breast cancer screening strategies: a systematic review and quality assessment. PLoS One 2019;14:e0226352. 10.1371/journal.pone.0226352 31841563 PMC6913984

[4] Oeffinger KC , Fontham ETH , Etzioni R , et al . Breast cancer screening for women at average risk: 2015 guideline update from the American cancer society. JAMA 2015;314:1599–614. 10.1001/jama.2015.12783 26501536 PMC4831582

[5] Shieh Y , Eklund M , Madlensky L , et al . Breast cancer screening in the precision medicine era: risk-based screening in a population-based trial. J Natl Cancer Inst 2017;109. 10.1093/jnci/djw290 28130475

[6] Kurian AW , Ward KC , Howlader N , et al . Genetic testing and results in a population-based cohort of breast cancer patients and ovarian cancer patients. J Clin Oncol 2019;37:1305–15. 10.1200/JCO.18.01854 30964716 PMC6524988

[7] Armstrong N , Ryder S , Forbes C , et al . A systematic review of the International prevalence of BRCA Mutation in breast cancer. Clin Epidemiol 2019;11:543–61. 10.2147/CLEP.S206949 31372057 PMC6628947

[8] Michailidou K , Lindström S , Dennis J , et al . Association analysis identifies 65 new breast cancer risk loci. Nature 2017;551:92–4. 10.1038/nature24284 29059683 PMC5798588

[9] Zhang H , Ahearn TU , Lecarpentier J , et al . Genome-wide Association study identifies 32 novel breast cancer susceptibility Loci from overall and subtype-specific analyses. Nat Genet 2020;52:572–81. 10.1038/s41588-020-0609-2 32424353 PMC7808397

[10] Torkamani A , Wineinger NE , Topol EJ . The personal and clinical utility of Polygenic risk scores. Nat Rev Genet 2018;19:581–90. 10.1038/s41576-018-0018-x 29789686

[11] Burton H , Chowdhury S , Dent T , et al . Public health implications from COGS and potential for risk stratification and screening. Nat Genet 2013;45:349–51. 10.1038/ng.2582 23535723

[12] Mavaddat N , Michailidou K , Dennis J , et al . Polygenic risk scores for prediction of breast cancer and breast cancer subtypes. Am J Hum Genet 2019;104:21–34. 10.1016/j.ajhg.2018.11.002 30554720 PMC6323553

[13] Willoughby A , Andreassen PR , Toland AE . Genetic testing to guide risk-stratified screens for breast cancer. J Pers Med 2019;9. 10.3390/jpm9010015 PMC6462925 30832243

[14] Apostolou P , Papasotiriou I . Current perspectives on Chek2 mutations in breast cancer. Breast Cancer (Dove Med Press) 2017;9:331–5. 10.2147/BCTT.S111394 28553140 PMC5439543

[15] Martin AR , Kanai M , Kamatani Y , et al . Clinical use of current Polygenic risk scores may exacerbate health disparities. Nat Genet 2019;51:584–91. 10.1038/s41588-019-0379-x 30926966 PMC6563838

[16] Wang S , Qian F , Zheng Y , et al . Genetic variants demonstrating flip-flop phenomenon and breast cancer risk prediction among women of African ancestry. Breast Cancer Res Treat 2018;168:703–12. 10.1007/s10549-017-4638-1 29302764 PMC5916755

[17] Allman R , Dite GS , Hopper JL , et al . SNPs and breast cancer risk prediction for African American and Hispanic women. Breast Cancer Res Treat 2015;154:583–9. 10.1007/s10549-015-3641-7 26589314 PMC4661211

[18] Shieh Y , Fejerman L , Lott PC , et al . A Polygenic risk score for breast cancer in US Latinas and Latin American women. J Natl Cancer Inst 2020;112:590–8. 10.1093/jnci/djz174 31553449 PMC7301155

[19] Behar DM , Yunusbayev B , Metspalu M , et al . The genome-wide structure of the Jewish people. Nature 2010;466:238–42. 10.1038/nature09103 20531471

[20] Carmi S , Hui KY , Kochav E , et al . Sequencing an Ashkenazi reference panel supports population-targeted personal Genomics and Illuminates Jewish and European origins. Nat Commun 2014;5:4835. 10.1038/ncomms5835 25203624 PMC4164776

[21] Delaneau O , Marchini J , Zagury J-F . A linear complexity phasing method for thousands of Genomes. Nat Methods 2011;9:179–81. 10.1038/nmeth.1785 22138821

[22] Howie BN , Donnelly P , Marchini J . A flexible and accurate genotype imputation method for the next generation of genome-wide association studies. PLoS Genet 2009;5:e1000529. 10.1371/journal.pgen.1000529 19543373 PMC2689936

[23] Purcell S , Neale B , Todd-Brown K , et al . PLINK: a tool set for whole-genome association and population-based linkage analyses. Am J Hum Genet 2007;81:559–75. 10.1086/519795 17701901 PMC1950838

[24] Chen Z-L , Meng J-M , Cao Y , et al . A high-speed search engine pLink 2 with systematic evaluation for Proteome-scale identification of cross-linked peptides. Nat Commun 2019;10:3404. 10.1038/s41467-019-11337-z 31363125 PMC6667459

[25] Choi SW , Mak T-H , O’Reilly PF . Tutorial: a guide to performing polygenic risk score analyses. Nat Protoc 2020;15:2759–72. 10.1038/s41596-020-0353-1 32709988 PMC7612115

[26] Purcell SM , Wray NR , Stone JL , et al . Common Polygenic variation contributes to risk of schizophrenia and bipolar disorder. Nature 2009;460:748–52. 10.1038/nature08185 19571811 PMC3912837

[27] Privé F , Vilhjálmsson BJ , Aschard H , et al . Making the most of Clumping and Thresholding for Polygenic scores. Am J Hum Genet 2019;105:1213–21. 10.1016/j.ajhg.2019.11.001 31761295 PMC6904799

[28] Privé F , Arbel J , Vilhjálmsson BJ . Ldpred2: better, faster, stronger. Bioinformatics 2021;36:5424–31. 10.1093/bioinformatics/btaa1029 33326037 PMC8016455

[29] Mak TSH , Porsch RM , Choi SW , et al . Polygenic scores via penalized regression on summary Statistics. Genet Epidemiol 2017;41:469–80. 10.1002/gepi.22050 28480976

[30] Dorling L , Carvalho S , Allen J , et al . Breast cancer risk genes — association analysis in more than 113,000 women. N Engl J Med 2021;384:428–39. 10.1056/NEJMoa1913948 33471991 PMC7611105

