# Supplementary material for: Evaluation of European-based polygenic risk score for breast cancer in Ashkenazi Jewish women in Israel
Source: J Med Genet. 2023 Jul 14;60(12):1186–97. doi: 10.1136/jmg-2023-109185 (PMC10715538; doi:10.1136/jmg-2023-109185)
Supplement: Supplementary data [file jmg-2023-109185supp001.pdf]

Supplementary Tables

Table S1. Performance of the PRS methods on the validation sets of the BCAC-IL cohort.

Method	OR per 1SD	Top 10% OR	# of SNPs
**P+T (EUR LD)**	1.48 ±0.04	2.33 ±0.32	1483±1230
**P+T (Target set LD)**	1.44 ±0.04	3.5 ±0.43	8591±12584
**LDPred2**	1.41 ±0.04	2.66 ±0.58	740801±41
**Lassosum**	**1.59 ±0.05**	**3.0 ±0.19**	65700±39569

In the nested CV scheme that we applied, in each split, the average OR per 1SD and the top 10% OR are computed over the validation sets (dark green in Figure 2). The last column is the average # of SNPs. Shown are means and standard deviations over the six splits.

Table S2. Performance of EUR PRS when excluding Polish and Israeli women from the discovery set and using these respective populations as the target cohorts.

Shown are means and standard deviations of the average performance obtained for the validation sets in each split (n=6).

Method	Target set cohort	OR per 1SD	Top 10% OR
P+T (EUR)	BCAC IL	1.42 ±0.03	**2.39 ±0.18**
	BCAC PL	**1.46 ±0.02**	2.11 ±0.08
P+T (Target set)	BCAC IL	1.39 ±0.03	**2.3 ±0.23**
	BCAC PL	**1.5 ±0.04**	2.14 ±0.12
LDPred	BCAC IL	**1.32±0.07**	1.61±0.09
	BCAC PL	1.17±0.03	**2.0±0.17**
Lassosum	BCAC IL	**1.54 ±0.04**	2.17±0.03
	BCAC PL	1.53 ±0.03	**3.13 ±0.24**

Levi H, *et al. J Med Genet* 2023; 60:1186–1197. doi: 10.1136/jmg-2023-109185

Supplementary Figures

Figure S1. PRS distribution of the holdout sets.

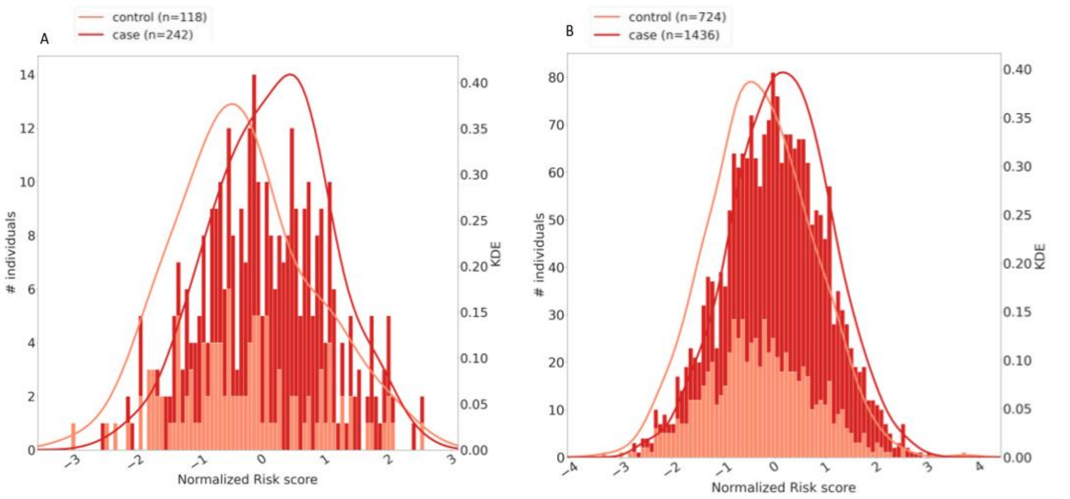

Scores were calculated using Lassosum with optimal hyperparameters. A: PRS on a single holdout set. B: Scores of all six holdout sets.

Levi H, *et al. J Med Genet* 2023; 60:1186–1197. doi: 10.1136/jmg-2023-109185

Figure S2. OR of BC risk as a function of BC-PRS percentiles on the validation sets.

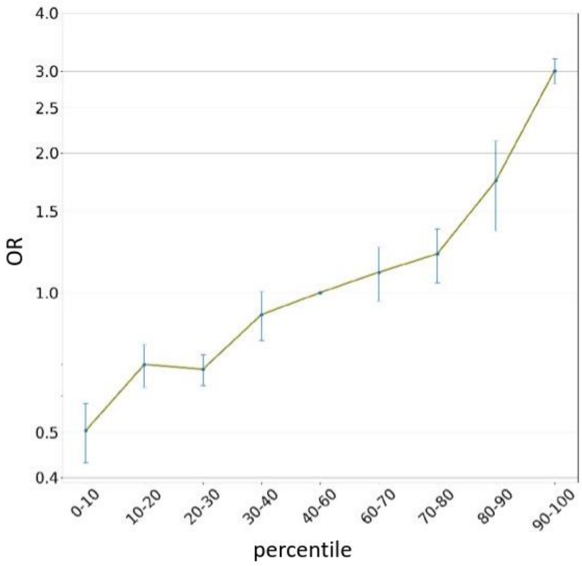

OR is measured relative to scores in the middle PRS quintile (40%-60%). PRS was generated by Lassosum. Shown are means and standard errors of the mean (SEM) over the average performance obtained on the validation sets in each split (n=6).

Figure S3. Performance of P+T EUR-LD PRS models on the BCAC-IL cohort with and without BRCA 1/2 carriers as a function of the threshold parameter (T).

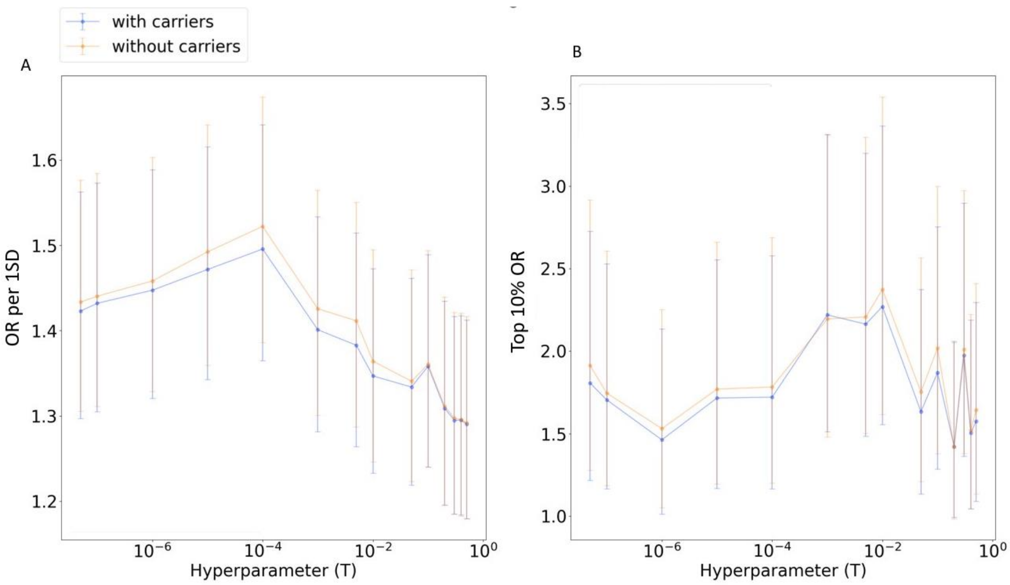

EUR GWAS was used as a discovery set. Performance measured by (A) OR per 1SD and (B) top-90% OR.

Levi H, *et al. J Med Genet* 2023; 60:1186–1197. doi: 10.1136/jmg-2023-109185

Figure S4. Outline of the generation of PRS model for HMC.

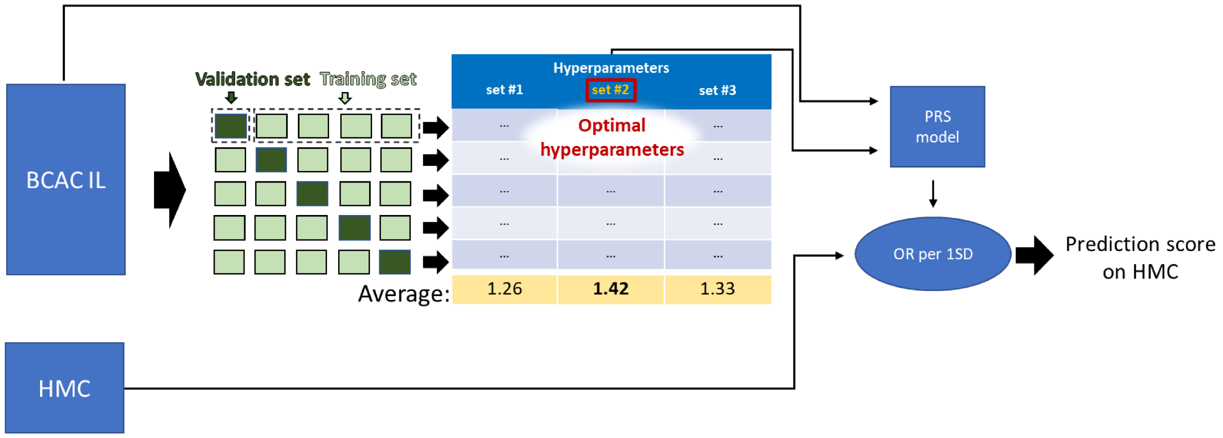

Levi H, *et al. J Med Genet* 2023; 60:1186–1197. doi: 10.1136/jmg-2023-109185

Figure S5. Average risk scores of patients in the BCAC-IL cohort stratified by age.

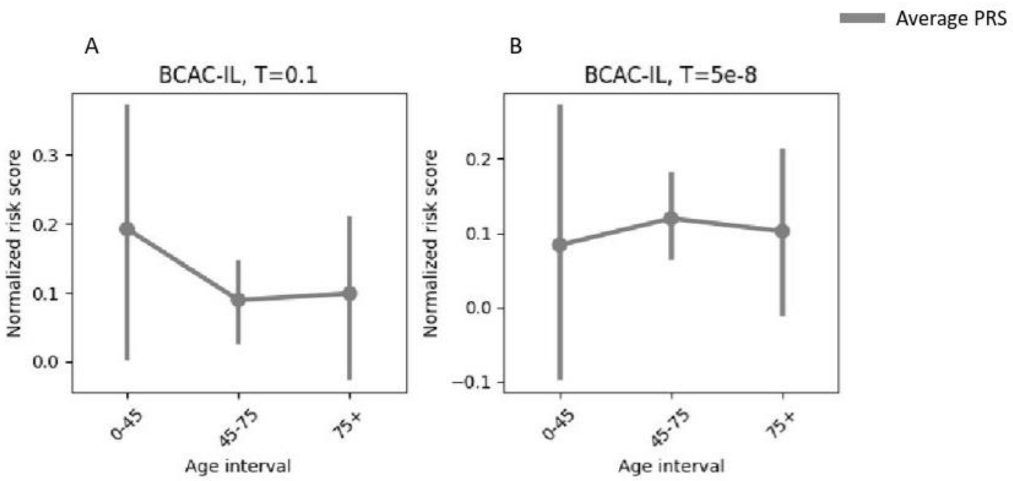

PRS obtained using GWAS of EUR individuals and the P+T EUR PRS method. P-value thresholds were (A) $T = 0.1$ and (B) $T = 5 \cdot 10^{-8}$.
